# Hydrogen Cyanamide Causes Reversible G2/M Cell Cycle Arrest Accompanied by Oxidation of the Nucleus and Cytosol

**DOI:** 10.3390/antiox12071330

**Published:** 2023-06-23

**Authors:** Yazhini Velappan, Ambra de Simone, Santiago Signorelli, John A. Considine, Christine H. Foyer, Michael J. Considine

**Affiliations:** 1 The UWA Institute of Agriculture, The University of Western Australia, Perth, WA 6009, Australia; 2 Centre for Plant Sciences, Faculty of Biology, University of Leeds, Leeds LS2 9JT, UK; 3 Food and Plant Biology Group, Departamento de Biología Vegetal, Facultad de Agronomía, Universidad de la República, Sayago CP 12900, Uruguay; 4 School of Biosciences, College of Life and Environmental Sciences, University of Birmingham, Edgbaston B15 2TT, UK; 5 Horticulture and Irrigated Agriculture, Department of Primary Industries and Regional Development, South Perth, WA 6151, Australia

**Keywords:** meristem, quiescence, cell cycle, redox, *roGFP2*, hydrogen cyanamide

## Abstract

Hydrogen cyanamide (HC) has been widely used in horticulture to trigger bud burst following dormancy. Its use has been banned in some countries due to human health concerns, however the search for effective safe alternatives is delayed by lack of knowledge of the mechanism of HC action. Earlier studies demonstrate that HC stimulates the production of reactive oxygen species (ROS) and alters the rate of cell division. However, the relationships between HC effects on ROS, redox (reduction/oxidation) homeostasis and cell division are unknown. This study used *Arabidopsis thaliana* ((L.) Heynh.) seedlings expressing the redox reporter *roGFP2* to measure the oxidation states of the nuclei and cytosol in response to HC treatment. The Cytrap dual cell cycle phase marker system and flow cytometry were used to study associated changes in cell proliferation. HC (1.5 mM) reversibly inhibited root growth during a 24 h treatment. Higher concentrations were not reversible. HC did not synchronise the cell cycle, in contrast to hydroxyurea. Rather, HC caused a gradual accumulation of cells in the G2/M phase and decline of G1/S phase cells, 16 to 24 h post-treatment. This was accompanied by increased oxidation of both the nuclei and cytosol. Taken together, these findings show that HC impairs proliferation of embryonic root meristem cells in a reversible manner through restriction of G2/M transition accompanied by increased cellular oxidation.

## 1. Introduction

Hydrogen cyanamide (HC, CAS no: 420-04-2) is widely used in the horticulture industry to trigger the resumption of growth following dormancy. While human health concerns have prompted review and restrictions on its use in some countries, the development of effective and safe alternatives is limited by knowledge of its mechanism of action. Effects of HC on the rate of cell division or gene markers thereof have been demonstrated in both roots and axillary buds [1,2,3,4]. In addition, HC is widely associated with triggering an oxidative burst of reactive oxygen species (ROS) [1,2,3]. In mammalian systems, ROS are required to initiate cell proliferation, and oscillations in ROS are coupled with cell cycle transitions [5,6]. It is worthwhile considering whether the effects of HC on the resumption of growth following dormancy in plants is dependent on ROS effects on cell division.

The cellular redox potential regulates cell biochemistry and hence plays a key role in tuning plant development to the local environmental conditions [7,8]. A network of interactions between reactive oxygen species, antioxidants, phytohormones and regulatory proteins act in a coordinated manner to control plant growth. In particular, changes in cellular redox status in response to external cues play an important role in regulating cell division in the root and shoot meristems [9,10,11]. The plant cell cycle comprises two principal phases of DNA synthesis (S phase) and mitosis (M phase), separated by two gap phases (G1 and G2, respectively) [12]. Progression through the cell cycle is regulated at particular checkpoints, notably the G1/S and G2/M transitions, depending on inter- and intra-cellular conditions and DNA fidelity. Inter-compartmental transport and sequestration of glutathione influences the transition of cells through key cell cycle checkpoints G1 and G2, by regulating the cellular redox state [9,10,11]. The nucleus and cytoplasm have similar levels of reduced glutathione (GSH) before entry into the cell cycle and this equilibrium is restored again during the G2-M phase of the cell cycle, with the redox state being highly regulated during the G1 and G2 checkpoints [9,10,11].

Roots are particularly vulnerable to direct interaction with phytotoxins and, as such, are excellent models of plant developmental plasticity in response to stress. Hence, the alteration of growth in roots in response to different oxidants and phytochemicals has been widely studied [2,3,13,14,15]. A recent study involving hydroxyurea (HU)-treated embryonic roots of *Arabidopsis thaliana* ((L.) Heynh.) established that progression of cells through the cell cycle is controlled by alterations in cellular redox homeostasis. The redox potential of both the nucleus and cytosol was determined using the redox-sensitive GFP (roGFP) reporter in *A. thaliana* seeds [11], and showed that depletion of the soluble antioxidant ascorbate disrupted the intracellular redox flux and rhythm of the cell cycle. In the present study, we adapted a similar approach to investigate the effect of HC on physiological growth and cellular redox homeostasis in relation to alterations in cell proliferation in embryonic roots of *A. thaliana*. The insight obtained from this study may later be transferred to other plant systems.

## 2. Materials and Methods

Unless otherwise stated, all chemicals were sourced from Sigma Aldrich (St. Louis, MO, USA).

### 2.1. Plant Material

*Arabidopsis thaliana* ((L.) Heynh.) wild-type (Col-0) seeds expressing redox sensitive green fluorescent protein (roGFP2; [16]), used to determine the redox state of the nuclei and cytosol in the embryonic root proliferation zone and wild-type (Col-0) seeds, were provided by Prof Christian Meyer (Institut Jean-Pierre Bourgin, Versailles, France). Col-0 seeds of a dual-core marker system (cell cycle tracking in plant cells; Cytrap) expressing pCYCB1::CYCB1-GFP and pHTR2::CDT1a (C3)-RFP [17], used to simultaneously monitor S/G2 and G2/M phases of the cell cycle, were obtained from Dr Masaaki Umeda (Nara Institute of Science and Technology, Ikoma, Japan).

### 2.2. Growth Conditions

Col-0 (wild-type), *roGFP2* and Cytrap seeds were surface-sterilised and transferred to plates containing half-strength Murashige and Skoog agar medium (½MS; [18]), prepared from 2.2 g·L^−1^ MS basal medium, 0.5 g·L^−1^ 4-Morpholineethanesulfonic acid, 0.1 g·L^−1^ Myoinositol, 10 g·L^−1^ sucrose and 10 g·L^−1^ agar at pH 5.7. The seeds were then stratified at 4 °C for 48 h and allowed to germinate at 21 °C in the dark for a further 48 h. The 2-d-old seedlings were transferred to fresh ½MS medium in the absence (control) or presence of 3 mM hydroxyurea (NH_2_CONHOH; HU, [11] or 1.5 mM HC (H_2_CN_2_) and grown at 21 °C in the dark until analysis (Supplementary Figure S1).

### 2.3. Root Growth Rate

Root length of Col-0 (wild-type) seeds was measured 0, 24, and 48 h after treatment with 3 mM HU and 1.5 mM HC, and in control seeds using ImageJ v1.51 image analysis software [19], and the root growth rate was calculated. Three biological replicates of 10 seeds each was used per time point for all the treatments.

### 2.4. Visualisation of Redox Status

**Figure 1:**
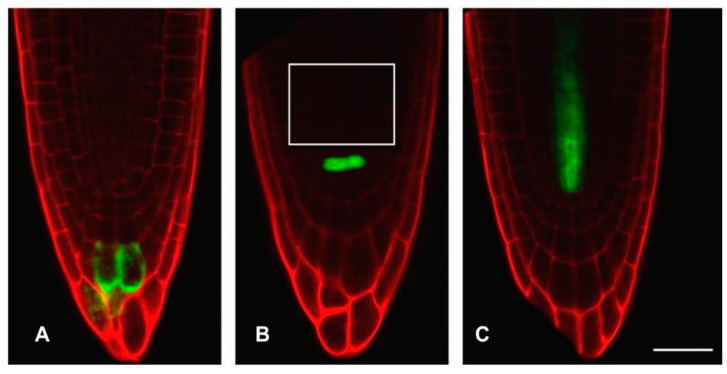
Distribution of GFP-tagged markers in the different cell types in *A. thaliana* roots. Root tips expressing GFP (green) tagged to (**A**) *PLETHORA* (*PLT3*) gene encoding AP2-domain transcription factors, (**B**) *WUSCHEL*-*related homeobox 5* (*WOX5*) (white box marks the proliferation zone) and (**C**) *WOODEN LEG* (*WOL*). Roots were stained with PI (red) on a microscope slide. Scale bar = 25 μm. Reproduced with permission from [11].

**Figure 2:**
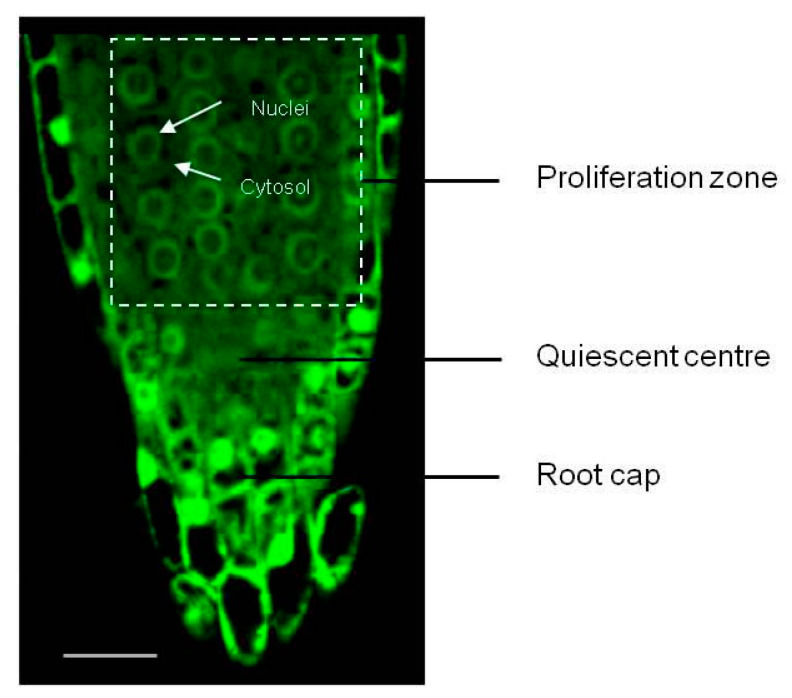
Root zones in *A. thaliana* embryonic root imaged using a confocal microscope at an excitation wavelength of 488 nm. Proliferation zone, quiescent centre and root cap cells in an *A. thaliana* embryonic root. The white arrows indicate nuclei and cytosol of a proliferation zone cell. Bar = 25 μm.

In the *A. thaliana* root meristem, the specification of individual cell types has been well-described, enabling the use of markers to spatially orientate meristematic zones [20]. The proliferation zone cells were identified based on the observations made by de Simone [21] using *PLETHORA 3* (*PLT3::GFP*), *WUSCHEL* (*WOX5::GFP*) and *WOODEN LEG* (*WOL::GFP*) markers to identify columella, quiescent centre (QC), and vascular system cells in embryonic roots of *A. thaliana* at the same developmental stage as used in this study (Figure 1 and Figure 2).

**Figure 3:**
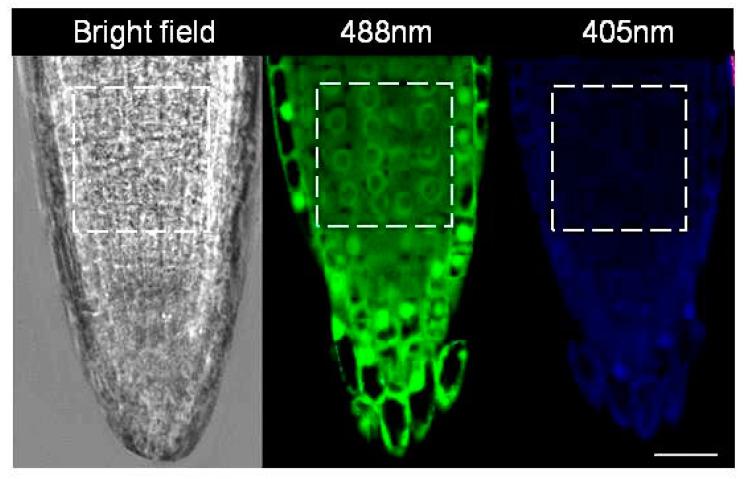
Confocal images of *A. thaliana* embryonic root tip in 2 d old *roGFP2* seedlings. The boxed area indicates the proliferation zone where fluorescence intensity measurements were carried out. Bar = 25 µm.

Degree of oxidation measurements, as explained further, were carried out every alternate hour (i.e., at 0, 2, 4 h…) over a period of 24 h after transfer to HC and control treatments, as described in the previous section. Germinated *roGFP2* seeds collected at various time points after treatment were placed on a drop of sterile water on a clean slide and imaged using the 40X/1.3 Oil DIC M27 lens of a LSM700 Carl Zeiss inverted confocal microscope (Carl Zeiss AG, Oberkochen, Germany) at excitation wavelengths of 405 nm for the oxidised and 488 nm for the reduced form of roGFP2 [11] (Figure 3). The degree of oxidation in the nuclei and cytosol of the cells in the proliferation zone above the quiescent centre was later determined from the ratio of the fluorescence intensities at 405 and 488 nm (405/488 nm), measured using ImageJ image analysis software [19]. Five technical and 5 biological replicates per time point were used in this study.

### 2.5. Calibration of roGFP2 Probe

The roGFP2 probes were calibrated at the end of the experiment, as described by de Simone et al. [11]. Briefly, embryonic roots were immersed in 2 mM dithiothreitol (DTT) solution for 10 min to cause complete reduction of roGFP2 before being imaged at excitation wavelengths of 405 nm and 488 nm. Similarly, to completely oxidise the roGFP2 probe, the embryonic roots were treated with 2 mM hydrogen peroxide (H_2_O_2_) solution for 15 min and later imaged at 405 nm and 488 nm, as shown in Figure 3. The 405/488 nm ratios calculated from these treatments were used for calibration of the roGFP probe during the calculation of the degree of oxidation and redox potential in the nuclei and cytosol of the cells in the proliferation zone of the embryonic root tip, as described in the next section.

### 2.6. Calculation of Redox Potential

Oxidation degree *(OxD_roGFP_*_2_) and redox potential of the *roGFP*2 (E_roGFP2_) probe were calculated as described by Meyer et al. [16], as follows:

(1) $$OxD_{roGFP2}=\;\frac{\left(R-R_{red}\right)}{\left(I_{488min}/I_{488max}\right)\left(R_{ox}-R\right)+\left(R-R_{red}\right)\;}$$

*OxD_roGFP_*_2_ was obtained using the roGFP2 fluorescence ratio (405/488 nm ratio) obtained using confocal microscopy as described previously, where *R* is the ratio of excitation (405/488 nm ratio), *R_red_* is the ratio of fully reduced roGFP2 obtained from 2 mM DTT treated embryonic root tip cells, *R_ox_* is the ratio of fully oxidised roGFP2 obtained from 2 mM H_2_O_2_ treated embryonic root tip cells. *I_488min_* and *I_488max_* are the fluorescence intensities of completely reduced and completely oxidised *roGFP*2, measured with excitation at 488 nm (Figure 3).

*OxD_roGFP_*_2_ obtained from the above equation was used to estimate the glutathione redox potential (*E_GSH_*) in mV using the following Nernst equation

(2) $$E_{roGFP2}=E_{roGFP2}^{O\prime }-\left(\frac{RT}{zF}\right)ln\left[\frac{1-OxD_{roGFP2}}{OxD_{roGFP2}}\right]$$

(3) $$E_{GSH}^{O\prime }-\left(\frac{RT}{zF}\right)ln\left[\frac{2{\left[GSH\right]}_{total}\;{\left(1-OxD_{GSH}\right)}^{2}}{OxD_{GSH}}\right]=E_{GSH}$$

where, *E* is the redox potential and *E^O′^* is the midpoint potential of GFP and *roGFP*2, *R* is the gas constant (8.315 J·K^−1^·mol^−1^), *T* is the absolute temperature (298.15 K), *z* is the number of electrons exchanged (2) and *F* is the Faraday constant (9.648 × 104 C·mol^−1^).

### 2.7. Cell Cycle Status

The cell cycle was monitored using the Cytrap system [17] and flow cytometry.

#### 2.7.1. Flow Cytometry

An intact nuclei suspension was prepared from fresh embryonic root tips using slight modifications to the protocol used by Arumuganthan and Earle [22]. Approximately 100 seeds per biological replicate (3 biological replicates) were used. Embryonic root tips of control and HC treated Col-0 wild-type seeds were carefully collected at 0, 5, 12, 16 and 24 h after treatment and chopped with a razor blade in ice cold nucleus-isolation buffer at pH 7.4 (10 mM MgSO_4_·_7_H_2_O, 50 mM KCl, 5 mM HEPES, 1 mg·mL^−1^ DTT, 0.5% (*v*/*v*) TritonX-100 and 1% (*w*/*v*) PVP-40) on ice and incubated on ice for 1 h with gentle swirling every 30 min. Following incubation, the suspension was passed through a 40 µm nylon mesh, centrifuged at 100× *g* for 10 min at 4 °C and the supernatant was carefully discarded. Later, the nuclei pellet was resuspended in 2 mL nucleus-isolation buffer and treated with RNase for 6 min at room temperature before being briefly stained with 20 µg·mL^−1^ propidium iodide and stored on ice until analysis. Stained cell samples were then run on a BD FACSCalibur flow cytometer (BD biosciences, Europe) at the Centre for Microscopy Characterisation and Analysis (CMCA), equipped with a primary blue 488 nm laser, and data for ~100,000 nuclei were recorded (i.e., until 20,000 G2 (4 °C) nuclei were collected). The proportion of nuclei with 2C and 4C DNA content was recorded. The data were visualised in real-time using scatter dot plots (FSC and SSC) and histograms. The results were analysed using Flowing Software version 2.5 (http://flowingsoftware.btk.fi/, accessed on 1 February 2018) by manual gating to eliminate debris from the population of interest on the scatter plots and subsequent generation of histograms from the scatter plot data in which the different populations were gated to obtain the final proportion of G1, S and G2 nuclei computed by the software. The data were plotted using Microsoft Excel 2016.

#### 2.7.2. Cytrap

**Figure 4:**
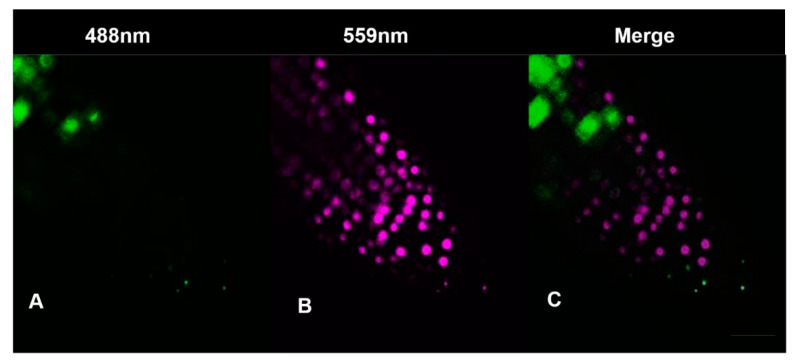
Confocal images of *A. thaliana* pHRT2::CDTa (C3)-RFP (magenta) and pCYCB1:: CYCB1-GFP (green) root tip obtained from excitation at 488 (GFP) and 559nm (RFP). (**A**) G2/M phase cells (green); (**B**) S/G2 phase cells (C3)-RFP (magenta); (**C**) Merge of A and B. Bar = 25 µm.

The Cytrap dual cell cycle phase marker system was used to monitor both the S/G2 and G2/M phases of the cell cycle simultaneously in proliferating cells of embryonic root [17]. The root tips of control and HC treated Cytrap seeds, placed in a drop of water on a clean slide, were imaged at 40× magnification using a LSM700 Zeiss inverted confocal microscope at excitation wavelengths of 488 nm for the green fluorescent protein (GFP; Figure 4A) and 559 nm for the red fluorescent protein (RFP; Figure 4B), at 0, 2, 5, 8, 10, 16, 18 and 24 h after treatment.

### 2.8. Data Analysis and Statistical Analysis

All calculations were performed and graphics were compiled using Microsoft Excel 2016. Significant differences among various sampling dates were corroborated statistically by applying a one-way ANOVA test, using Tukey’s honestly significant difference (HSD) post hoc test with *p* ≤ 0.01 and *p* ≤ 0.05 (Origin; OriginLab, Northampton, MA, USA).

## 3. Results

### 3.1. Hydrogen Cyanamide Causes a Reversible Effect on Embryonic Root Growth of A. thaliana

HC is phytotoxic at high concentrations [2,3,23]. At low concentrations, however, HC relieves dormancy and promotes growth in perennial buds [23,24]. Although several studies have implicated the involvement of redox regulation in dormancy release by HC, the regulation of growth by HC has not been studied at the cellular level. Hence this study was designed to examine the influence of HC on cell proliferation and redox state. A dose-response experiment was first carried out to determine the concentration of HC that caused reversible arrest of root growth on short-term treatment without having any phytotoxic effect, analogous to the 3 mM HU concentration used in an earlier study, to determine the effect of cellular redox regulation on the status of the cell cycle in embryonic root meristems [11].

**Figure 5:**
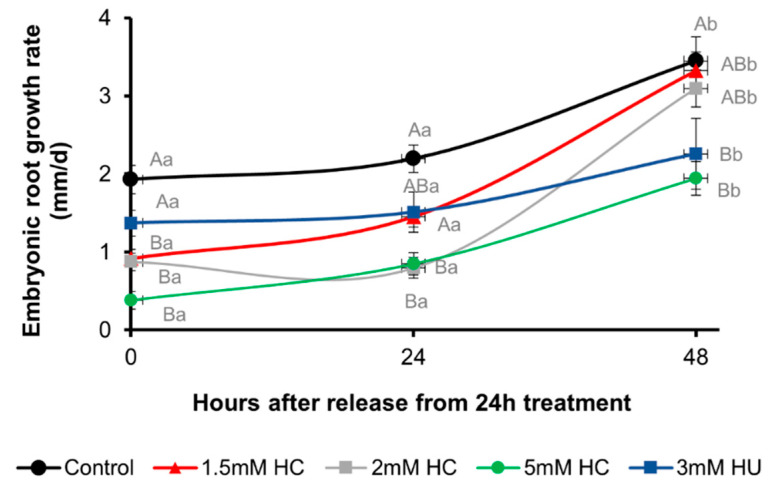
Recovery of root growth after release from treatment in control, HC and HU treated *A. thaliana* seedlings. Rate of root growth in control, 1.5, 2 and 5 mM HC and 3 mM HU treated seedlings, 0, 24 and 48 h after recovery from 24 h treatment in the dark at 21 °C. Lower case letters above bars denote significant differences (*p* ≤ 0.01) within the same treatment at different time points and upper case letters above bars denote significant differences (*p* ≤ 0.01) at the same time point among different treatments using Tukey’s honestly significant difference (HSD) test.

The concentration of HC was optimised. Two-day-old seedlings grown in the dark at 21 °C were treated with a range of concentrations of HC (1.5, 2 and 5 mM) or 3 mM HU for 24 h under the same conditions, and later released from the treatment by transferring them to HC or HU free media and grown for a further 48 h at 21 °C in the absence of light. The corresponding control seedlings were also grown under similar conditions. Root length measurements were made immediately after transfer from treatments (0 h), and at 24 h and 48 h after transfer to control media (Figure 5).

Treatment with 1.5 mM HC and 3 mM HU for 24 h caused a reduction in root growth rate compared to the control. Upon transfer to control media, roots treated with 1.5 mM HC and 3 mM HU had a growth rate of *ca* 45% and 72% of the control roots (Figure 5). After 24 h of recovery from these treatments, both 1.5 mM HC and 3 mM HU treated roots recovered >50% of the control root growth rate. After 48 h recovery, the growth rate of 1.5 mM HC treated roots was comparable to the control (Figure 5). However, HU treated roots only recovered 65% of the control root growth. Higher concentrations of HC (2 mM and 5 mM) retarded the growth rate further than that of the 1.5 mM HC treated roots, such that the 5 mM HC treated roots did not recover the control growth rate within the 48 h period.

The concentration of 1.5 mM HC was chosen for further studies because we intended to study cell division and oxidation within a 24 h period, and this minimal concentration posed the least likelihood of pleiotropic effects due to phytotoxicity.

### 3.2. Hydrogen Cyanamide Decreases Root Growth of A. thaliana Seedlings in a Time-Dependent Manner

**Figure 6:**
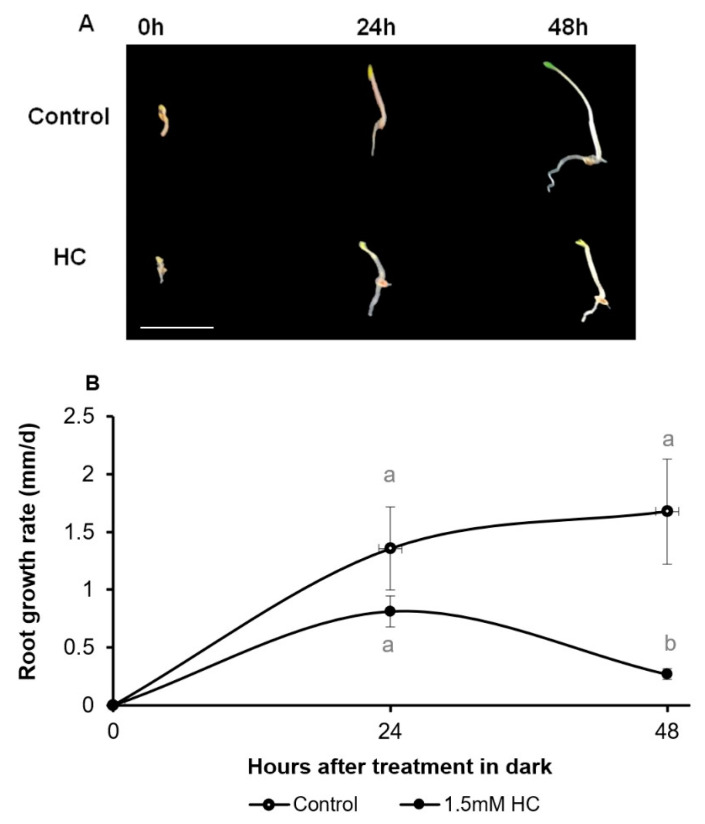
Root growth in control and HC treated *A. thaliana* seedlings. (**A**) Macroscopic images of control and HC treated seedlings after 0 h, 24 h and 48 h of treatment. Bar = 1 cm. (**B**) Rate of root growth in control (open circles), 1.5 mM HC (closed circles) treated seedlings after 24 and 48 h of treatment in the dark at 21 °C. Lower case letters above bars denote significant differences (*p* ≤ 0.01) at same time point among different treatments, corroborated using Tukey’s honestly significant difference (HSD) test. There was no significant difference at *p* ≤ 0.01 within the same treatment at different time points.

Following the optimisation of HC concentration and studying the dose-dependent effect of HC, the time-dependent effect of 1.5 mM HC on root growth in comparison to the control was studied. Two-day-old seedlings germinated in the dark at 21 °C were transferred to ½MS media supplemented with and without 1.5 mM HC and grown for 48 h under the same growth conditions. Root growth was measured immediately after the transfer (0 h) (Figure 6), 24 h and 48 h after the transfer (24 h and 48 h respectively) (Figure 6). Roots of control (untreated) seedlings grew well during the whole treatment period (Figure 6A), maintaining a uniform growth rate of 1.4 and 1.7 mm·d^−1^ by 24 and 48 h after treatment, respectively (Figure 6B). By comparison, seedlings treated with HC displayed a progressively reduced rate of growth, with their growth rate approximately halving between 24 and 48 h.

### 3.3. Hydrogen Cyanamide Treatment of Embryonic Root Tips Causes Gradual Accumulation of G2/M Phase Cells

**Figure 7:**
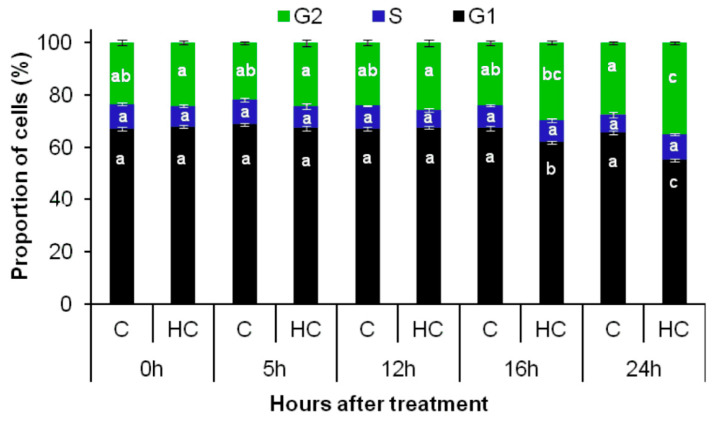
Distribution of cells in each cell cycle phase in control and HC treated root tips. Proportion of cells in G1, S and G2 phases of the cell cycle in control (C) and cyanamide (HC) treated *A. thaliana* embryonic root tip cells at various time points of treatment. Lower case letters below the bars denote significant differences (*p* ≤ 0.01) in the distribution of cells within the same cell cycle phase among different time points of control and HC treatment, corroborated using Tukey’s honestly significant difference (HSD) test.

**Figure 8:**
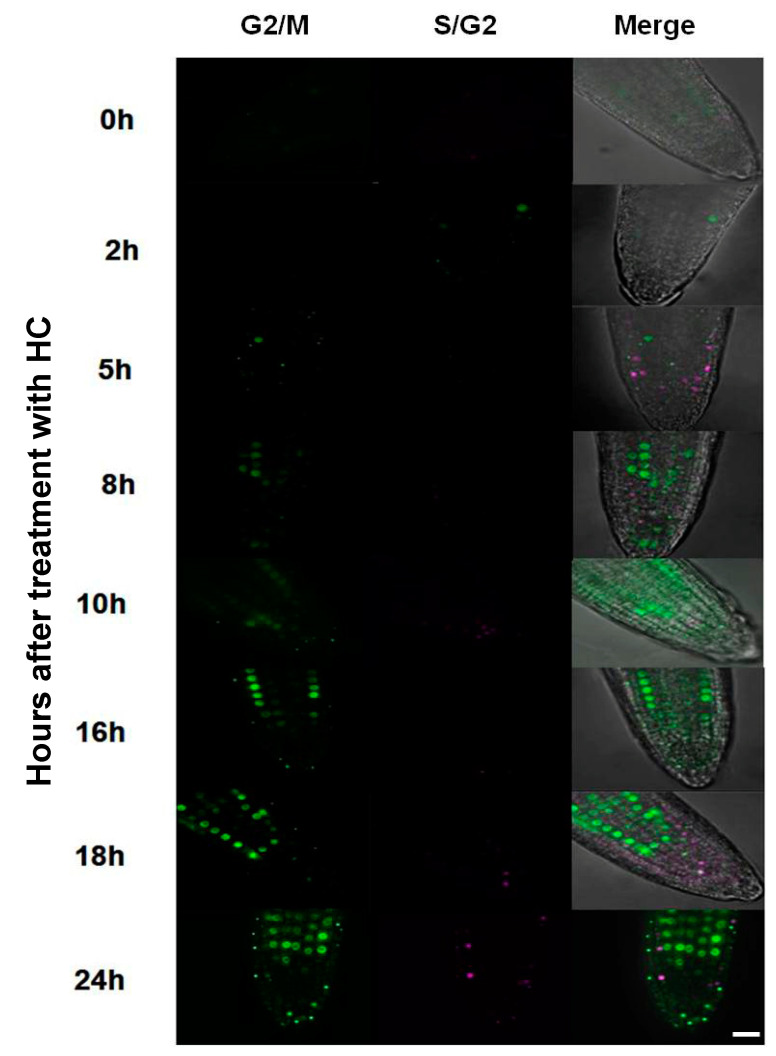
Cytrap expression in control and HC treated *A. thaliana* embryonic root tip cells at various time points of treatment. The expression pattern in the control remained the same at all time points and similar to 0 h treatment of HC. Hence, a common representative figure for the control and 0 h HC treatment is shown here. Magenta and green fluorescence shows distribution of cells in S/G2 (Phtr2:: CDT1a (C3)-RFP expression) and G2/M (Pcycb1:: CYCB1-GFP expression) phase of the cell cycle, respectively, and merge shows overlay of S/G2 and G2/M with a bright-field image background. Bars = 25 µm. Only one representative figure out of 3 replicates for each time point is shown. The weak signal of Phtr2:: CDT1a (C3)-RFP (S/G2) reflects low numbers of cells in that phase (Figure 7).

Cell cycle progression was monitored both in vitro and in vivo in the absence and presence of HC in *A. thaliana* embryonic root tips using flow cytometry and the Cytrap marker system. Untreated control root tips maintained around 67% G1, 8% S and 25% G2 cells during 24 h of treatment, showing no significant change in the proportion of cells in each phase of the cell cycle (Figure 7). Untreated root tips of Cytrap seedlings showed undetectable fluorescence when excited at 488 nm (G2/M phase) and 559 nm (S/G2 phase) during the 24 h of treatment (Figure 8 and Figure S2), indicating the asynchronous nature of cell cycle progression in the proliferation zone of control root tips. However, there was a significant difference in the cell cycle status of HC treated root tip cells from 16 to 24 h of treatment. The proportion of cells in the G1 phase showed a significant decrease, from >65% during the earlier time points to 55% at 24 h. This decrease in G1 cells was accompanied by a considerable increase in G2 phase cells at 24 h (8%) of HC treatment (Figure 7). Similarly, HC treated root tips of Cytrap seeds showed a gradual increase in fluorescence when excited at 488 nm (G2/M phase) from 16 h to 24 h of HC treatment (Figure 8). These results indicate an accumulation of G2/M phase cells after 16 h of HC treatment, with a corresponding decline in G1/S phase cells (Figure 7 and Figure 8). However, no change was detected in the S/G2 (magenta coloured) channel during the 24 h of HC treatment compared to the control (Figure 8), or in the distribution of S phase cells (Figure 7). Taken together, these data suggest that treatment of *A. thaliana* embryonic root tips with 1.5 mM HC for 24 h prolongs the cell cycle at the G2/M phase. However, these data require further validation through analysis of the expression pattern of cell cycle related genes at various time points after treatment, with HC in comparison to the untreated control.

### 3.4. Hydrogen Cyanamide Triggers a Higher Cellular Oxidation Compared to Hydroxyurea

Fluorescence ratios determined in the nuclei and cytosol of embryonic root proliferation zone cells of germinating *A. thaliana roGFP2* seeds treated with 1.5 mM HC and 3 mM HU and the untreated control were used to calculate the degree of oxidation, which in turn was used to calculate glutathione redox potentials. Untreated control and HU treated root proliferation zone cells had similar mean glutathione redox potentials of −296.8 ± 0.9 and −297.4 ± 1.1 mV in the nuclei and −294.1 ± 1.4 and −295.2 ± 1.6 mV in the cytosol, respectively, over 24 h of treatment (Table 1). However, the nuclei and cytosol of HU treated cells were relatively less oxidised (21% in the nuclei and 24% in the cytosol) compared to the control cells (23% in the nuclei and 26% in the cytosol) (Table 1). The HC treated cells were most oxidised during the 24 h period of treatment, with glutathione redox potentials of −291.4 ± 1.9 and −289.6 ± 1.4 mV and oxidation degrees of 31% and 32% in the nuclei and cytosolic compartments, respectively (Table 1). On average, HC treated cells were 5–10% more oxidised than the HU treated and control cells (Table 1). Moreover, cytosol was rather more oxidised than the nuclei in the embryonic root proliferation zone cells, irrespective of the treatment (Table 1).

### 3.5. Accumulation of G2/M Cells Is Accompanied by Increased Oxidation in Hydrogen Cyanamide Treated Root Tips

**Figure 9:**
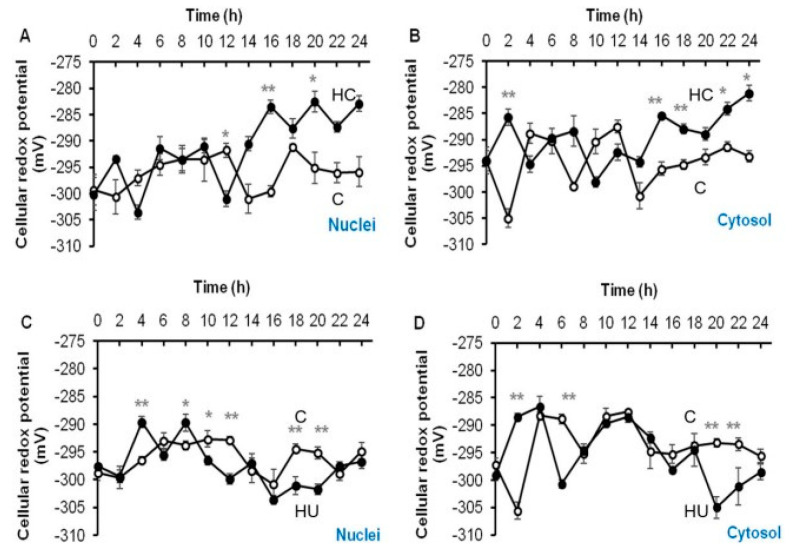
Changes in cellular redox potential occurring in the proliferating cells of embryonic root of germinating *A. thaliana* seeds. Effect of HC (**A**,**B**) and HU (**C**,**D**) treatment on cellular redox potential of meristematic zone cells, in *roGFP2*-expressing roots, during 24 h of treatment. Fluorescence was measured in the absence (C, Control, open circles) or presence (filled circles) of treatment with HC and HU. * and ** above bars denote significant differences (* *p* < 0.05, ** *p* < 0.01) in comparison to the untreated control, using Tukey’s honestly significant difference (HSD) test.

**Figure 10:**
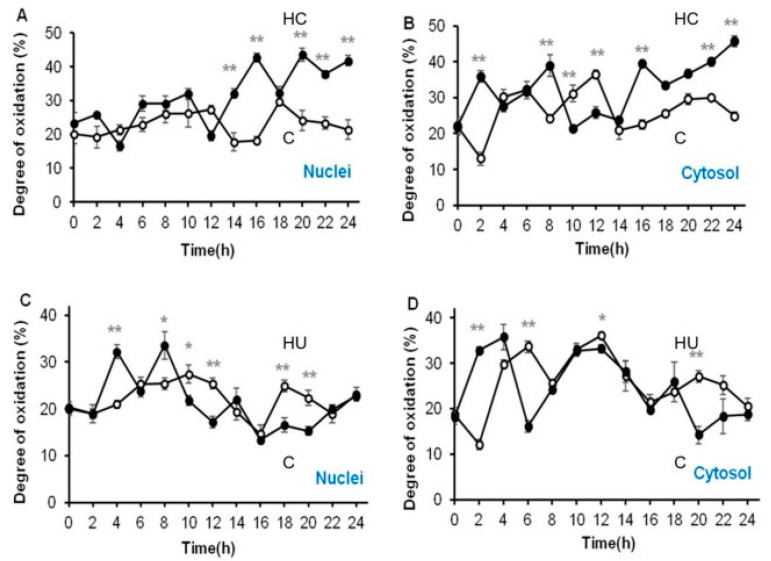
Changes in cellular oxidation occurring in the proliferating cells of embryonic root of germinating *A. thaliana* seeds. Effect of HC (**A**,**B**) and HU (**C**,**D**) treatment on degree of oxidation of meristematic zone cells, in *roGFP2*-expressing roots, during 24 h of treatment. Fluorescence was measured in the absence (C, Control, open circles) or presence (filled circles) of treatment with HC and HU. * and ** above bars denote significant differences (* *p* < 0.05, ** *p* < 0.01) in comparison to the untreated control, corroborated using Tukey’s honestly significant difference (HSD) test.

Treatment with HC triggered an oxidation event in the cytosol immediately after treatment (2 h), increasing the redox potential to −285.74 ± 1.1 mV and oxidation degree to 37%, respectively, in comparison to the untreated control that had a much lower redox potential of <−300 mV and oxidation degree of nearly <20% in both nuclei and cytosol (Figure 9A,B and Figure 10A,B). HU treatment caused a similar increase in redox potential and oxidation degree in the cytosol (−288.53 ± 0.7 mV and 32%, respectively) 2 h after treatment, but only after 4 and 8 h of treatment in the nuclei (*ca* −289 mV and 32% respectively) (Figure 9C,D; [11]). There was no significant change in redox potential or oxidation degree of the nuclei until 12 h post-treatment with HC (Figure 9A,B and Figure 10A,B). During the first 8 h of treatment, most of the cells in the proliferation zone of HU synchronised root tips were considered to be in the G1/S phase (Figure 9C,D; [11,14]). No such synchronisation of the cell cycle was observed in HC treated root tip cells (Figure 8).

Redox potential of HC treated cells varied from −303 to −282 mV in the nuclei and −298 to −281 mV in the cytosol (Figure 9A,B). The HU treated cells remained comparatively less oxidised, with nuclei and cytosolic redox potentials ranging from −303.60 to −289.64 mV and 304.997 to 286.64 mV, respectively (Figure 9C,D and Figure 10C,D). The nuclei and cytosol of the control were relatively more reduced than those of HC but not HU treated cells (Table 1). The redox state of nuclei and cytosol of HC treated cells did not differ significantly in comparison to the control in the first 12 h of treatment (Figure 9A,B and Figure 10A,B). However, after 12 h of treatment with HC, the nuclei and cytosol became highly oxidised, as opposed to the significantly reduced untreated control cells during this period (Figure 9A,B and Figure 10A,B). The degree of oxidation in the nuclei and cytosol remained greater than that of the control in HC treated cells, as opposed to HU treated cells that remained more reduced than the control in the final hours of treatment (12–24 h; Figure 10), when the HU synchronised cells were considered to be in G2 and M phases of the cell cycle [11,14] and HC treated cells showed a progressive increase in G2/M cells (Figure 7 and Figure 8). Overall, a higher degree of oxidation was observed in both the cellular compartments studied, during the early hours of HU treatment when G1 and S phase cells were predominant. Conversely, a lower degree of oxidation was observed during the final hours of HU treatment, when the majority of cells accumulated in the G2 and M phases of the cell cycle [11].

HC caused a gradual accumulation of G2/M cells in the later hours of the 24 h HC treatment (Figure 8). HC stimulated a higher degree of oxidation in the final hours of treatment, parallel to the accumulation of G2/M cells, in addition to an early oxidation event 2 h after HC treatment (Figure 7, Figure 8 and Figure 10).

## 4. Discussion

HC is an allelochemical commonly used in agriculture to relieve bud dormancy and promote growth [23,24,25]. It is known to act through the generation of ROS, mainly H_2_O_2_, leading to alterations in redox homeostasis associated with the oxidation of glutathione (GSH to GSSG), which may regulate cell proliferation [1]. However, if and how HC alters cell proliferation through associated changes in the cellular redox state is unknown. The complexity of the meristems in other organs makes it difficult to study the mode of action of HC at the cellular level. Hence, this study used the *A. thaliana* embryonic root system to study the effect of HC on the cell cycle and redox at the cellular level.

Regardless of its widespread use as an agrochemical, the effectiveness of HC largely depends on the concentration of application, with higher doses being detrimental to growth [2,3,26,27]. In this study, a 1 d treatment of *A. thaliana* seedlings with 1.5 mM HC caused ca 40% reduction in root growth rate compared to the untreated control, consistent with the changes observed in roots of tomato (*Solanum lycopersicum* L.) treated with 1.2 mM HC [2], onion (*Allium cepa* L.) treated with 2 mM HC [3], maize (*Zea mays* L.) treated with 3 mM HC [28] and lettuce (*Lactuca sativa* L.) treated with 10 µM HC [29]. Moreover, continuous treatment of *A. thaliana* seedlings with the same concentration of HC for 2 d caused an 84% decline in root growth rate compared to the untreated control in this study. Interestingly, tomato roots treated with 1.2 mM HC only showed a 50% reduction in root growth rate compared to the control after 3 d of treatment [2], similar to 3 mM HC treated maize roots [28]. This difference in sensitivity to HC could be attributed to the relatively thinner root system of *A. thaliana*, thus an increased surface area to volume ratio.

Prolonged treatment (48 h) with 1.5 mM HC caused a decrease in root growth rate compared to the short-term treatment (24 h), which is similar to observations made in tomato, in which shrinkage of only root tips but not the distal root segments was observed after 3 d of HC treatment [2]. The authors ascribed this to earlier cellular differentiation following the end of mitosis, rather than to variations in cell length. The same could be true for *A. thaliana* roots in this study. However, this needs further validation. The effects of short-term HC treatment on root growth rate were completely reversible at the 1.5 mM concentration, which is similar to previous observations in tomato roots treated with 1.2 mM HC [2]. However, *A. thaliana* roots recovered to control levels 2 d after release from treatment, as opposed to tomato roots, which required 5 d to reach control levels [2]. This could perhaps be due to the difference in growth medium, physiological state of the seedlings or their ability to recover from stress. Onion roots treated with similar concentration of HC (2 mM) for a short period showed increased growth after recovery from treatment, indicating the growth promoting effect of HC at low dosage [3]. Similar growth promoting effects were also observed in lettuce roots treated with low concentrations of rabdosin B [15]. However, such induction of growth was not observed in this study at the lowest concentration used (1.5 mM). This could be because this concentration was not low enough to have a growth promoting effect. Therefore, the inhibitory effect of HC in *A. thaliana* root growth is dose- and time-dependent, with its effect being more pronounced and irreversible at higher doses and/or when treated for longer durations, which is analogous to earlier observations [2,28].

*A. thaliana* seedlings treated with 3 mM HU for a short period did not show significant reduction in root growth rate compared to the control, which is in agreement with earlier studies [21]. HU had a slightly higher root growth rate compared to 1.5 mM HC treated seedlings during short-term treatment. However, 2 d after release from treatments, only roots treated with 1.5 mM HC were able to fully recover their growth to untreated control levels. The previous study by de Simone [21] did not observe the recovery effect of 3 mM HU, however no phytotoxicity was evident, based on staining for cell viability. Hence, this delay in recovering control levels of growth, even in the absence of phytotoxicity, needs to be explored further.

Earlier studies report that HC mediated reduction in root growth is caused by perturbations in the division of cells at the meristematic region [2]. In this study, short-term treatment with 1.5 mM HC (24 h) caused significant alterations in cell cycle status only from 16 h of treatment, as opposed to HU, which alters cell cycle status immediately after treatment, causing an accumulation of S phase cells [14,21]. HC treatment caused a gradual build-up in the population of G2 phase cells, with an accompanying decline in G1 phase cells, implying a gradual decline in dividing cells, which is similar to observations made in tomato roots treated with 1.2 mM HC for 3 d [2]. This is in contrast to the observation in onion roots treated with 2 mM HC, which did not show any changes in the distribution of cells in various cell cycle phases after short-term treatment [3]. This result was further supported by observations using the Cytrap marker system, which indicated an accumulation of G2/M phase cells from 16 h until the end of treatment. However, the decline in the G1 population observed using flow cytometry, could not be verified by this marker system. Moreover, no significant change in the S/G2 phase was detected during the 24 h HC treatment. In addition, an earlier study reported that HU treated roots showed a clear increase in S/G2 cells 5–10 h after treatment [21]. This effect of HU is due to synchronisation of the cell cycle by a transient G1/S arrest, as reported by Cools et al. [14]. The replication restriction imposed by HU in the earlier study was overcome within the first 5–6 h of treatment, commencing the first cycle of DNA replication following synchronous progression to the S phase after HU treatment, which is similar to results observed by Cools et al. [14]. In contrast, HC has a delayed and cumulative effect on cell proliferation from 16 h post-treatment. This study did not observe the effect of HC beyond 24 h. Investigation of the effect of HC on root meristem cells for a longer duration may improve the understanding of how HC affects the cell cycle. Overall, HC affects cell proliferation in a different manner to HU, by blocking the G2/M transition and inhibiting mitosis.

Intracellular redox is highly regulated at the major cell cycle checkpoints, G1 and G2, to ensure proper progression of cells through the cell cycle [11]. Inter-compartmental transport and sequestration of the antioxidant glutathione is important in modulating the cellular redox state [11]. Treatment with HC in this study triggered an oxidation event in the cytosolic compartment immediately post-treatment, causing an increase in redox potential, similar to the effect observed on HU treatment [21]. In previous studies, this was shown to be due to the transport of GSH into the nucleus from the cytosol during the G1 phase, leading to depletion of the cytosolic GSH pool, indicated by the higher degree of oxidation in the cytosol compared to the nuclei [9,10]. The HU treated cells were predominantly in the G1 phase at this time period [14,21]. HC treatment did not cause any significant alteration in the redox potential of the nucleus in the early hours of treatment. During the later hours of the treatment, when G2/M phase cells began to accumulate, both the nucleus and the cytosol were highly oxidized, compared to the control and in contrast to HU treated cells, which were maintained in a reduced state [14,21]. The accumulation of cells at the G2/M checkpoint at the later points of HC treatment may be due to the increased oxidation, which depleted the cellular GSH pool, causing a GSH deficiency and altering the levels of CYCs and CDKs necessary for G2/M transition. This has been observed in cucumber roots treated with 0.25 mM phenylcarboxylic acid, which caused inhibition of *CYCB* gene expression [13,30,31].

## 5. Conclusions

The nuclei and cytosol of proliferation zone cells in *A. thaliana* radicles are maintained in a highly reduced state and have similar glutathione redox potentials. HC treatment triggered an oxidative stress towards following ca 16 h of treatment, which was accompanied by G2/M phase cell cycle arrest. This arrest could be due the depletion of the total cellular GSH pool, causing significant oxidation in both the nuclei and the cytosol. This study advances understanding of the mode of action of hydrogen cyanamide in plant growth.

## Figures and Tables

**Table 1:** Glutathione based average cellular redox potential and oxidation degree for control (untreated), HC (cyanamide) and HU (hydroxyurea) treated proliferation zone cells of *A. thaliana* embryonic roots over a 24 h treatment period. (Data represent mean values ± SEM; n ≥ 3).

	Redox Potential (mV)			Degree of Oxidation (%)		
	Control	HC	HU	Control	HC	HU
Nuclei	−296.8 ± 0.9	−291.4 ± 1.9	−297.4 ± 1.1	22.90 ± 2.3	31.22 ± 1.6	21.35 ± 1.3
Cytosol	−294.1 ± 1.4	−289.6 ± 1.4	−295.2 ± 1.6	26.31 ± 1.6	32.48± 1.4	24.44 ± 1.8

## Data Availability

Data are contained within the manuscript and supplementary materials.

## Supplementary Materials

The following supporting information can be downloaded at: https://www.mdpi.com/article/10.3390/antiox12071330/s1, Figure S1: Schematic showing the experimental design; Figure S2: Cytrap expression in control *A. thaliana* embryonic root tip cells at various time points of treatment.
