# Supplementary material for: Hydrogen Cyanamide Causes Reversible G2/M Cell Cycle Arrest Accompanied by Oxidation of the Nucleus and Cytosol
Source: Antioxidants (Basel). 2023 Jun 23;12(7):1330. doi: 10.3390/antiox12071330 (PMC10376265; doi:10.3390/antiox12071330)
Supplement: Supplementary file 1 [file antioxidants-12-01330-s001.zip › antioxidants-2408708-supplementary.pdf]

Supplementary Figures

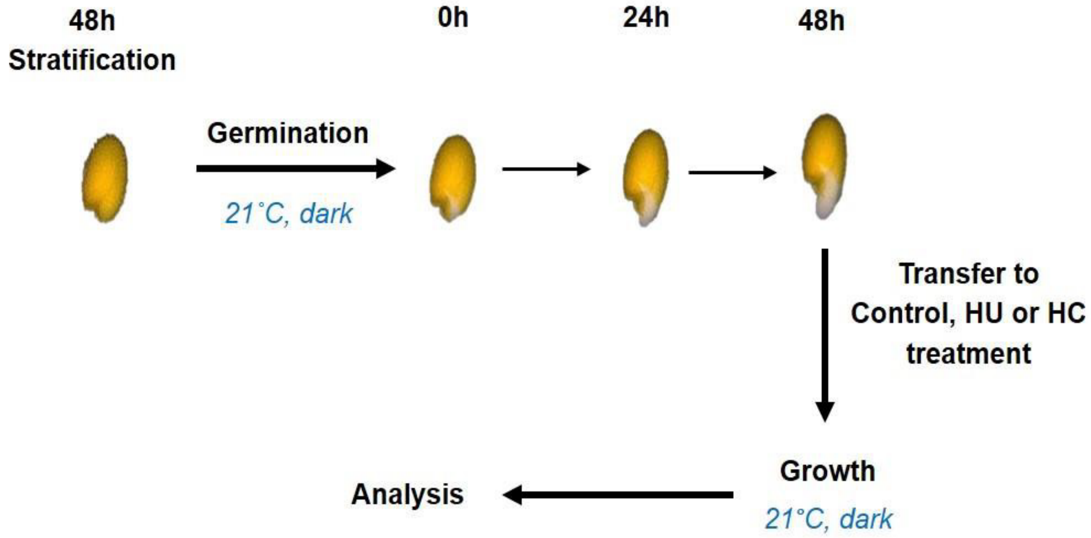

Supplementary Figure S1. Schematic showing the experimental design. roGFP2 and Cytrap seeds were stratified for 48hr in the dark at 4˚C and later allowed to germinate at 21˚C under dark condition for 48hr, followed by transfer to chemical treatment (3mM HU or 1.5mM HC) or control conditions for root growth measurements, *in vivo* measurement of redox state and monitoring cell cycle status in the proliferation zone of the embryonic root. HC- Hydrogen cyanamide, HU- Hydroxy urea.

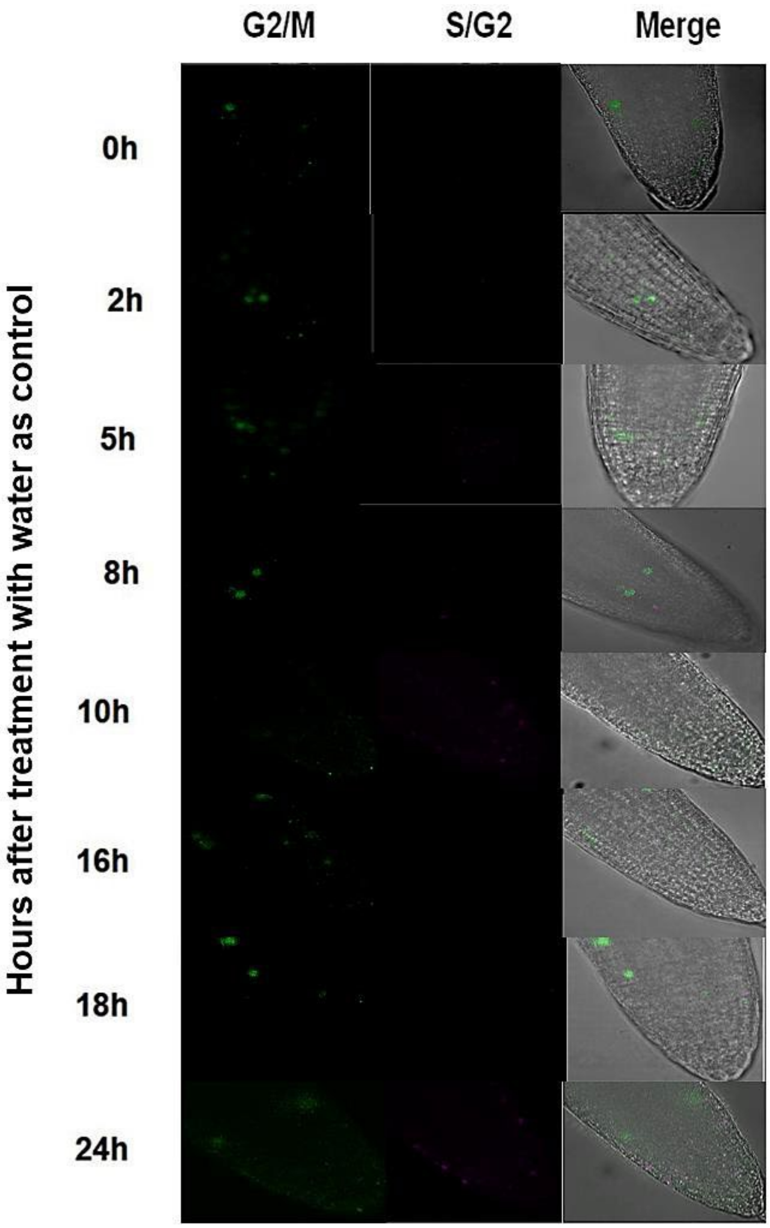

Supplementary Figure S2. Cytrap expression in control Arabidopsis embryonic root tip cells at various time points of treatment. The expression pattern in the control remained the same at all time points. Magenta and green fluorescence shows distribution of cells in S/G2 (Phtr2::CDT1a (C3)-RFP expression) and G2/M (Pcycb1:: CYCB1-GFP expression) phase of the cell cycle respectively and merge shows overlay of S/G2 and G2/M with a bright-field image background. Bars= 25µm. Only one representative figure out of 3 replicates for each time point is shown.
